# Mineral status of soil, sea water, and mangrove (*Avicennia marina*) forages in several coastal areas of West Sumatra

**DOI:** 10.14202/vetworld.2021.1594-1601

**Published:** 2021-06-21

**Authors:** Gusri Yanti, Novirman Jamarun, Suyitman Suyitman, Benni Satria, Rani Winardi Wulan Sari

**Affiliations:** 1 Department of Animal Science, Faculty of Animal Science, Andalas University, Kampus Limau Manis, Padang, West Sumatera, Indonesia; 2 Department of Animal Nutrition, Faculty of Animal Science, Andalas University, Kampus Limau Manis, Padang, West Sumatera, Indonesia; 3 Department of Agrotechnology, Faculty of Agriculture Andalas University, Kampus Limau Manis, Padang, West Sumatera, Indonesia

**Keywords:** *Avicennia marina*, forage, mineral content, soil

## Abstract

**Background and Aim:**

The availability of minerals in the soil affect the mineral content of mangrove leaves. This study aimed to determine the macro- and micromineral contents in the environment and mangrove leaves (*Avicennia marina*) as animal feed in the coastal areas of West Sumatra, Indonesia.

**Materials and Methods:**

In this study, soil, water, and mangrove leaves were extracted from the mangrove plant’s environment. The mineral contents were determined using the atomic absorption spectrophotometer of Beijing Rayleigh Analytical Instrument Corporation (make and country of origin). The total phenol and tannin contents were determined using the Folin–Ciocalteu and hide-powder methods, respectively.

**Results:**

The mineral content of the soil affected the mineral content of the plants. The soil and leaves of *A. marina* in the Pariaman area were richer in terms of macro- and microminerals. The soil had pH value, organic carbon content, leaf nitrogen content, phosphorus, calcium, and potassium of 5.65, 4.21%, 3.39%, 0.17%, 1.99%, and 0.54%, respectively. *A. marina* leaves had a total phenol and tannin contents of 24.51 mg GAE/g check the unit and 4.09%, respectively.

**Conclusion:**

This research showed that the mineral content in the soil in several mangrove areas in West Sumatra has a positive correlation with the mineral content in the leaves of *A. marina*, which have a complete mineral content. Therefore, *A. marina* leaves could be recommended as a mineral source for ruminants.

## Introduction

The coastal area of West Sumatra, such as that in South Pesisir, is a productive location for the growth of ruminants. However, the lack of quality natural forage has led breeders to import feed ingredients from outside the region. This does not only reduce the efficiency of feed costs but also farmers in these locations are forced not to raise livestock owing to the challenge in finding forage and the limited quantity. Apart from the coastal area in Pesisir Selatan, the surrounding communities in the Padang Pariaman coast contain buffalo herds. Furthermore, the limited number of grasses acting as forages makes livestock development least attractive. The mangrove plants around the coastal area are considered to possess the potential to become forages. Furthermore, in Pakistan’s Indus delta, approximately 16,000 camels and 11,000 cows eat mangrove leaves [1]. Each year, during the start of the flood season (June–July), camels from the remote areas of Sindh migrate to the mangrove area in herds and stay until October [2].

Based on this study, mangroves (*Avicennia marina*) have the potential to be a substitute for forage in ruminants. *A. marina* has this potential due to its high micro- and macromineral contents, which is required by livestock. However, the growth of a mangrove plant is largely determined by environmental conditions, such as water, soil, and climate, which support its growth [3]. These results yielded differences in micro- and macromineral contents.

The difference in micro- and macromineral contents in soil and water will significantly influence the same mineral in *A. marina* leaves. All leaves depend on soil and water for the supply of mineral nutrients, and ruminants derive most of their mineral nutrients from forage plants eaten. The concentration of mineral elements in forages depends on the interaction of several factors, such as soil, water, plant species, maturity level, yield, soil management, and climate [4]. Moreover, mineral deficiency in ruminants is directly related to soil and water characteristics [5].

This study aimed to determine the macro- and micromineral contents in the environment and mangrove leaves (*Avicennia marina*) as animal feed in the coastal areas of West Sumatra, Indonesia. The results will serve as a reference to map the potential of *A. marina* and determine the best place for *A. marina* leaves that contain macro- and microminerals needed to feed ruminants. Furthermore, it contributes to the fulfillment of the forages requirement of farmers around mangrove locations.

## Materials and Methods

### Ethical approval

This study does not need ethical approval as it is not related to animals.

### Study period and location

**Figure-1:**
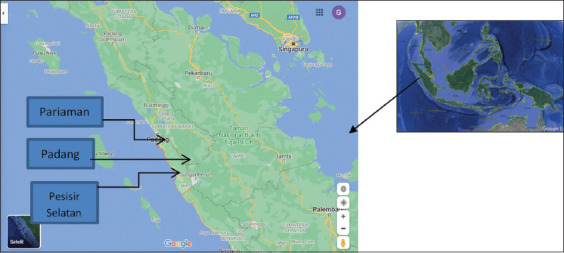
Map of research location in West Sumatera Province, Indonesia [Source: www.maps.google.com].

The study was conducted in August 2020. The area of mangrove forests in Indonesia ranges between 2.5 and 4.5 million hectares. West Sumatra Province has a coastal area of about 186,500 sq.km and a coastline length of 1973.24 km (Figure-1). The area of mangrove forests in West Sumatra is around 39,832 ha spread across the regencies of Mentawai 32,600 ha, Pasaman 6,273.5 ha, Pesisir selatan 325 ha, Agam 313.5 ha, Padang Pariaman 200 ha, and Padang 120 ha. Samples were processed at Water Laboratory of Technical Industry Department, Technic Faculty, Andalas University.

### Sample collection

Soil, water, and mangrove (*A. marina*) leaf samples were obtained from several places in the coastal areas of West Sumatra, with three locations, including Regency of Padang, Pariaman, and Pesisir Selatan. These samples were collected at ten points in each area (soil, water, and *A. marina* leaves) and composited. Soil samples were obtained using a stainless steel sampling auger, with a depth of 15 cm. In addition, a forage subsample was collected from each region by taking a few plots from each *A. marina* tree (such as taking on tea leaves). Soil and leaf samples were then dried in an oven at 60°C for 48 h, milled using a mill, and filtered using a 1-mm stainless steel sieve for forage or a 2-mm sieve for soil. The water sample that had been obtained was filtered using Whatman No 1 (GE Healthcare, China) and subsequently stored in a bottle and labeled.

### Mineral analysis

A total of 1 g of soil and leaf samples were processed with sulfuric acid and hydrogen peroxide in a flask following the wet digestion method. Soil and forage samples were filtered and added with distilled water up to 50 mL. The filtered sample was then stored in a bottle and labeled and placed in the ­laboratory. The mineral content in the soil, water, and forage samples was determined using the atomic absorption spectrophotometer method/tool of the Beijing Rayleigh Analytical Instrument Corporation after wet digestion. The digestion of wet process was performed by adding sulfuric acid to the sample and heating it on a dry block until the sample turned black. Hydrogen peroxide with 30% concentration was heated until the solution turned clear, diluted until 100 mL, and filtered using Whatman No. 41 (GE Healthcare).

Nitrogen (N), phosphorus (P), and chlorine (Cl) were measured using the Kjeldahl method, ultra violet (UV)-visible (Shimadzu) spectrophotometer with a wavelength of 889 nm, and titration method, respectively; other minerals were measured using the atomic absorption spectrophotometer with a wavelength of potassium (K, 766.5 nm), calcium (Ca, 422.7 nm), magnesium (Mg, 285.2 nm), sulfur (S, 666 nm), iron (Fe, 248.3 nm), copper (Cu, 324.7 nm), manganese (Mn, 279.5 nm), zinc (Zn, 213.9 nm), molybdenum (Mo, 460 nm), mercury (Hg, 253.7 nm), and lead (Pb, 283.3 nm).

### Analysis of the total phenol and tannin of mangrove leaves (*A. Marina*)

#### Total phenol (Folin–Ciocalteu method)

A total of 0.5 g powder sample was mixed with a 1-mL 0.5% hexamethylenetetramine, 20-mL acetone, and 2-mL 25% hydrochloric acid (HCl). The mixture was kept in a reflow refrigerator for 30 min and shaken with distilled water and ethyl acetate. The extract was separately used in the two solutions. In the first part, the extract was mixed with 1-mL aluminum chloride and 5% (volume/volume) methanol-acetic acid for further measurement. Meanwhile, the second part was mixed with 5% (volume/volume) methanol-acetic acid to produce a standard solution. After 30 min of incubation, the absorbance of both samples was measured at 425 nm (A). The total phenolic concentration was calculated using the formula: (1.25×A) m, where m = mass of sample in grams.

#### Total tannin (hide-powder method)

The mangrove (*A. marina*) leaves, which had been mashed to 0.5 g, were mixed with 150 mL of distilled water and heated in a water bath for 30 min at 70°C. The cooled extract was then quantitatively transferred into a 250-mL volumetric flask, filtered, and used for the reaction. The results of the extraction were measured using the UV-visible (Shimadzu) spectrophotometer at a wavelength of 278.5 nm using pure tannins as standard.

### Statistical analysis

The results were analyzed using generalized linear models method with IBM SPSS Statistics 26.0 version (IBM Corp., NY, USA).

## Results

Soil pH values and organic carbon (C-org) content in the coastal areas of West Sumatra are shown in Table-1. The C-org content and pH values were shown to be significantly different (p<0.05) and vary for each coastal area of West Sumatra.

**Table-1:** C-organic content and pH value of soil in coastal areas of West Sumatra.

Variable (%)	Soil		

	Padang	Pariaman	Pesisir Selatan
pH	5.25^a^±0.07	5.65^b^±0.06	5.15^a^±0.07
C-org	3.54^a^±0.13	4.21^b^±0.09	3.60^a^±0.11

^a,b^Superscript different means significantly different in a row (p<0.05)

The macromineral content of soil, sea water, and *A. marina* leaves (Padang, Pariaman, and Pesisir Selatan) is shown in Tables-2-4. The macromineral contents of each region were shown to be significantly different (p<0.05).

**Table-2:** Macromineral content (percent dry) in growth areas (soil) in several coastal areas of West Sumatra.

Variable (%)	Padang	Pariaman	Pesisir Selatan
N	2.72^a^±0.02	3.00^c^±0.014	2.84^b^±0.057
P	0.32±0.04	0.34±0.013	0.29±0.007
Ca	2.29^b^±0.01	2.43^b^±0.014	2.10^ab^±0.014
K	0.41^a^±0.106	0.49^b^±0.014	0.41^a^±0.014
Mg	1.03^b^±0.021	0.90^a^±0.021	0.97^ab^±0.019
S	0.01±0.007	0.02±0.007	0.01±0.007

^a,b,c^Different superscripts mean significant difference in a row (p<0.05)

**Table-3:** Macromineral content in growth areas (water) in several coastal areas of West Sumatra.

Variable (%)	Padang	Pariaman	Pesisir Selatan
N	9.82^a^±0.028	10.61^b^±0.078	11.84^c^±0.163
P	0.81^a^±0.021	0.77^a^±0.021	1.13^b^±0.028
K	13.45^a^±0.035	14.45^b^±0.269	15.01^b^±0.021
Ca	4.13 ±0.036	4.81±0.212	4.47±0.092
Mg	3.91^a^±0.071	4.23^b^±0.049	4.60^c^±0.021
S	1.09^a^±0.028	1.32^b^±0.048	1.63^c^±0.085

^a,b,c^Different superscripts mean significant difference in a row (p<0.05)

**Table-4:** Macromineral content *Avicennia marina* leaves in several coastal areas of West Sumatra.

Variable (%)	Padang	Pariaman	Pesisir Selatan
N	3.08^a^±0.106	3.49^b^±0.078	3.05^a^±0.049
P	0.14±0.028	0.17±0.021	0.17±0.028
K	1.83^a^±0.026	1.99^b^±0.035	2.04^c^±0.014
Ca	0.38^a^±0.064	0.54^b^±0.028	0.69^c^±0.028
Mg	0.87^b^±0.049	0.15^a^±0.014	0.81^b^±0.021
S	0.005±0.001	0.009±0.001	0.007±0.001

^a,b,c^Different superscripts mean significant difference in a row (p<0.05)

The micromineral composition of soil, sea water, and *A. marina* leaves (Padang, Pariaman, and Pesisir Selatan) is shown in Tables-5-7. The micromineral contents of each region were shown to be significantly different (p<0.05). The composition of heavy metals (Hg and Pb) in soil, sea water, and *A. marina* leaves (Padang, Pariaman, and Pesisir Selatan) is shown in Table-8. The content of heavy metals (Hg and Pb) in the soil was shown to be significantly different (p<0.05), whereas that in the water and *A. marina* leaves were not significantly different.

**Table-5:** Micromineral content in soil in several coastal areas of West Sumatra.

Micro minerals (ppm)	Padang	Pariaman	Pesisir Selatan
Fe	6.53^a^±0.064	8.06^b^±0.078	8.89^c^±0.127
Cu	4.18^a^±0.035	8.86^b^±0.076	13.38^c^±0.035
Mn	15.85^a^±0.064	28.03^c^±0.035	20.92^b^±0.198
Zn	13.89^a^±0.021	14.43^a^±0.064	20.20^b^±0.014
Mo	3.40^c^±0.035	2.06^a^±0.042	2.39^b^±0.134
Cl	1.01^b^±0.028	0.90^a^±0.028	0.81^a^±0.035

^a,b,c^Different superscripts mean significant difference in a row (p<0.05)

**Table-6:** Micromineral content sea water in several coastal areas of West Sumatra.

Micro minerals (ppm)	Padang	Pariaman	Pesisir Selatan
Fe	0.33±0.049	0.30± 0.035	0.34±0.028
Cu	0.04^a^±0.006	0.07^b^± 0.006	0.08^a^±0.007
Mn	0.05^a^±0.002	0.1^ab^± 0.003	0.14^c^±0.016
Zn	0.086±0.005	0.115± 0.074	0.149±0.021
Mo	0.081±0.004	0.090± 0.005	0.111±0.069
Cl	135.88^a^±0.120	154.11^b^± 0.032	239.50^c^±0.064

^a,b,c^Different superscripts mean significant difference in a row (p<0.05)

**Table-7:** Micromineral content in *Avicennia marina* leaves in several coastal areas of West Sumatra.

Micro minerals (ppm)	Padang	Pariaman	Pesisir Selatan
Fe	1.00^a^±0.021	1.18^b^±0.028	1.19^b^±0.014
Cu	0.49±0.127	0.67±0.064	0.89±0.007
Mn	1.05^a^±0.035	1.23^b^±0.049	1.65^c^±0.035
Zn	2.02^a^±0.021	2.27^b^±0.035	2.52^c^±0.057
Mo	0.41±0.028	0.43±0.021	0.47±0.028
Cl	0.75±0.035	0.71±0.085	0.57±0.057

^a,b,c^Different superscripts mean significant difference in a row (p<0.05)

**Table-8:** Heavy metal content in soil, sea water and *Avicennia marina* leaves in several coastal areas of West Sumatra.

Variable (ppm)	Padang	Pariaman	Pesisir Selatan
Soil			
Hg	0.42^a^±0.021	0.57^b^±0.021	0.67^c^±0.007
Pb	0.98±0.062	1.22±0.049	0.93±0.035
Water			
Hg	0.0036±0.001	0.0040±0.005	0.0028±0.012
Pb	0.0083±0.002	0.0094±0.007	0.0089±0.028
*Avicennia marina* leaves			
Hg	0.08±0.021	0.04±0.003	0.07±0.021
Pb	0.23±0.007	0.24±0.005	0.25±0.171

^a,b,c^Different superscripts mean significant difference in a row (p<0.05)

## Discussion

### Macromineral content of soil, water, and leaves of *A. marina*

The pH and C-org content of the soil where *A. marina* grows is shown in Table-1. The soil pH is 5.15-5.65, which is acidic because it originates from swampy areas affected by tidal water. The mangrove soil is muddy and rich in alluvial deposits, clay, humus, nonorganic salt content, Ca, S, Fe, and Mn, which influence its aroma and dark color. According to Heriyanto and Suharti [6], the acidity (pH) of this soil is approximately 5. The soil pH is strongly influenced by the C-org content. The highest C-org content (4.21%) was found in the Pariaman soil. Based on physical observations, this soil is darker and contains several organic matter derived from mangrove litter and the weathering of dead roots. The organic matter is another factor that greatly affects soil pH conditions. During decomposition, the organic matter produces organic acids that reduce the pH of the soil and its environment, including the surrounding water [7].

According to Gordon [8], the mangrove area is defined as a dynamic place where mud and land are continuously formed by plants that slowly turn into semi-terrestrial areas (semi-land). Soil (sediment) that is formed serves as a place for living organisms to thrive and find food. The fertility of mangrove sediments is due to the organic material they contain. The organic material is one of the components of the water-based substrate, which consists of heaps of plant and animal remains. Furthermore, mangrove ecosystems are tropical and subtropical coastal vegetation communities dominated by several species of mangrove trees that grow and develop in tidal muddy coastal areas [9]. These vegetation formers are tree species that adapt physiologically to relatively high salinity, delicate soil structure, composition, and influence from tides.

In the Pariaman area, the soil N content ranged from 2.7% to 3%, which is significantly different from the Padang and South Pesisir mangrove areas. The increase in the total soil N content corresponds with the C-org content in the soil. It occurs due to the decomposition of organic matter, where N is the main element derived. The decomposition process is largely dependent on the quality and amount of organic material. According to Hugues *et al*. [10], one of the factors influencing the rate of decomposition is an increase in the soil pH. A decrease toward the neutral will increase the activity of soil microorganisms. Since the activity of microorganisms increases in the soil, the C/N ratio is also decreased; therefore, N is available to plants.

The factors of soil and plants that affect forage mineral content have been widely reported [11]. In plants, the mineral content is strongly influenced by the quantity and availability of minerals in the soil. Availability is very significant because, from several minerals, only a small portion can be absorbed. Furthermore, the process of soil mineral decomposition is influenced by the origin of the source rock, surface erosion, application of fertilizers, and waste materials. Their availability in the soil and uptake by plants depends on factors, such as pH, moisture, and drainage conditions.

N is the main food for plants as it is a constituent of protein and chlorophyll, which activates the photosynthesis process. N has the most significant role in various physiological processes, such as giving dark green color to plants, increasing the number of leaves, stems, and others. In addition, it influences rapid growth and increases the protein content of plants. This function greatly determines the quality of the *A. marina* leaves as a forage. When the data content of N (3%) is converted into crude protein, the crude protein content in *A. marina* leaves in the coastal area of West Sumatra is approximately 20%. This content is high enough to meet the protein requirement of ruminants. According to Ghosh *et al*. [12], the crude protein content *A. marina* leaves is estimated to be 15%, and this also encourages the absorption and utilization of other nutrients, including P and Ca, for overall plant growth.

The dissolved P content is an indicator that determines the fertility level of the water. In this research location, the sea water was classified in the normal category due to the P level of 0.7-1.1%. This was also influenced by the condition of mangrove stands in the location. P typically appears in low concentrations in natural sea water due to a large amount of mobility, although the total phosphate concentration often ranges between 0.01 mg/L and >200 mg/L [13].

P in soil, sea water, and mangrove plants vary in amounts, although not statistically significant. The P content in the soil is approximately 0.3%, 0.7-1.1% in sea water, and 0.1% in *A. marina* leaves. P is essential for plant growth and is found in every living plant cell. It is involved in photosynthesis, respiration, energy storage and transfer, cell division, and enlargement. The results showed that P is an important component of the photosynthetic process, systematically involved in the formation of sugars, oils, and starches that further aids in the conversion of solar to chemical energy, proper maturation of plants, and the ability to withstand stress. Moreover, ruminants require a large amount of P elements. The adequacy of P in ruminants is obtained from forage containing it. In addition, P plays a significant role in the development and metabolism of microorganisms in the rumen [14]. Its deficiency results in reproductive disorders, such as anesthesia, low conception, long calving intervals, embryo death, and delayed sexual maturity [15].

The Ca content in mangrove leaves ranged between 1% and 2%. The forage originating from South Pesisir regency contains the highest Ca content of 2.04%. This content is able to meet the Ca requirement of ruminants. Approximately 99% of the total Ca in the body functions as a structural component of bones and teeth. The remaining 1% is involved in vital functions, such as blood clotting, membrane permeability, neuromuscular stimulation, secretion of certain hormones, and activation of enzymes. The lack of Ca results in soft and weak bones and decreases growth and milk production [5]. Ca and P have a very close relationship with each other in terms of metabolic processes in animals. Furthermore, the balanced nutritional value of Ca and P depends on the adequacy of the supply of each feed source, the ratio, and the presence of Vitamin D. The ideal Ca and P ratio is between 2:1 and 1:1 [12].

The Mg content in mangrove leaves (*A. marina*) ranged between 0.1% and 0.9%. This content is high and adequately meets the requirement of ruminants. The function of Mg, in general, includes cofactors of >300 enzymes that function in carbohydrate, fat, and protein metabolism [16], form ribosomes, and maintain membrane integrity through bonding with phospholipids [17]. Furthermore, it is required in membrane energy transport, cyclic adenosine monophosphate formation and transmission of genetic material, muscle contraction, nerve transmission, and constitutes the main components of bone structure [18]. The normal Mg percentage in the body is 65-70%, 15%, 15%, and 1% in bones, muscles, soft tissues, and extracellular fluid, respectively[19].

The K content in mangrove leaves ranged between 0.3% and 0.7%. This value considerably varies and is highest in South Pesisir. For forage, in general, K and Cl have a similar function. K maintains the osmotic pressure, controls water balance, and regulates acid-base balance. Furthermore, it functions in muscle contraction and nerve impulse transmission, whereas Cl is required for the formation of HCl in the stomach. The signs of Cl and K deficiency are nonspecific and reduce feed intake, growth, and milk production [4]. Muscle weakness, including the uterine muscles, occurs due to K deficiency, which indirectly causes reproductive problems by inhibiting the use of protein and energy. This results in weakness of the uterine muscles, thereby increasing the risk of metritis and placental retention[20]. Furthermore, the provision of excessive amounts of K (5% dry matter) is believed to cause delayed puberty and ovulation, impaired corpus luteum development (yellow body), and an increased incidence of anestrus in cattle[21].

The macromineral content in the coast of West Sumatra was relatively varied and sufficient to meet the mineral needs of ruminants. According to Maxwell and Lai[22], *A. marina* is used as a candidate for mineral nutrition supplementation to increase milk production in dairy cows in New Zealand. According to Sathe *et al*. [23], this is due to the high nutritional content and suitability for replacing grass or plant sources that are frequently used as animal feed. However, the content of P and Ca in *A. marina* has a significant comparison with some feed substances, such as *Oryza sativa* or *Arachis hypogea*.

### Micromineral content of soil, water, and mangrove leaves

The results of the micromineral content analysis in sea water, soil, and mangrove leaves are shown in Table-3. The value of Fe contained in the soil around mangrove plants is relatively high. The Fe content in each region was shown to be significantly different, with the highest content found in South Pesisir at 8.89 ppm. Furthermore, the high Fe^2+^ values can be explained by the anaerobic conditions of mangrove soils, which favor greater dissolution in this environment. This condition is equally supportive in increasing the amount of Mn^2+^ and related to the amount of organic matter in the soil. Conversely, Zn^2+^ is expressed in high-enough concentrations, as a negative response to aerobic conditions during tidal and soil drying processes. Moreover, it is associated with carbon dioxide accumulation from the decomposition of organic matter and pH fluctuations, which ­according to Numbere and Camilo[24], allows the precipitation of zinc carbonate, zinc hydroxide, and zinc sulfide to occur.

In general, the micromineral requirements of livestock are relatively varied. These levels will affect the quality of mangrove leaves (*A. marina*) as forage. The Zn content in forage was observed to range from 2.00 to 2.52 mg/g, whereas the Zn requirement for ruminants is 10-50 mg/kg. When there is a Zn deficiency status, the rumen microbial activity is not optimal. Therefore, the level of feed utilization will be lower, which in turn, will reduce livestock productivity [5]. Zn deficiency in ruminants, results in the loss of weight, appetite, and hair, with skin lesions on the legs, neck, head, and around the nose, excessive salivation, and reproductive problems. Zn deficiency in males leads to a decreased testicular development and sperm production, whereas in females, it causes cyclical disorders and conception rates [25].

Zn is an essential micromineral for growth, reproduction, immune system, gene expression, enzyme processes, performance, and health of livestock[17]. Furthermore, it functions in livestock metabolism and involves the regeneration of keratin and epithelial tissue integrity, bone metabolism, nucleic acid synthesis, cell division, protein synthesis, catalytic ions, structure and regulation of enzymes and transcription factors, participating in carbohydrate, fat, and protein metabolism, sexual development and spermatogenesis, immune function, and appetite control through its action on the central nervous system. Zn also participates in the structural, catalytic, and regulatory functions of most species[26].

The Cu levels in *A. marina* mangrove leaves were not significantly different in each region, as it ranged between 0.4 and 0.8 ppm, whereas the Cu requirements for ruminant is 15-20 mg/kg. Cu is an essential micronutrient in all living organisms, from plants to mammals, and is needed in very small amounts. This element plays a significant role in various physiological functions of the hematological, nervous, cardiovascular, reproductive, and immune systems [27]. Furthermore, it is a vital microelement in the pigmentation of mammalian skin and hair. Tyrosinase, a Cu-containing enzyme (the enzyme required for the conversion of tyrosine to melanin) is very important for the pathway of pigmentation. Therefore, decreased tyrosinase concentrations and depigmentation in ruminants are observed as early signs of hypocuprosis or Cu deficiency[28].

The Mn content ranged between 1.2 and 1.6 ppm in *A. marina* leaves. The Mn requirement for ruminants is 0.20-0.60 mg/kg. Mn from leaves originating from the South Coast is relatively high at 1.6 ppm. This content is sufficient to meet the Mn requirement of ruminants. According to Mousavi *et al*. [29] stated that Mn mineral deficiency rarely occurs because the Mn content in feed is sufficient for livestock needs. Mn minerals are needed in small amounts, although have a very significant function. According to Hansen *et al*. [30], its deficiency in livestock will result in dwarfism.

The Mo content of *A. marina* leaves in each region was not significantly different at 0.4 ppm, since the normative Mo requirement in ruminants is <100 mg/kg^1^. A high amount of Mo suppresses Cu availability and causes physiological Cu deficiency in ruminants. Total S or sulfate in the ration generally supplements the Mo effect. The Cu to Mo ratio in feed is important regardless of the absolute number of each. Therefore, in consideration of the significance of dietary S content, it is not possible to establish food safe limits for Cu and Mo. Physiological Cu deficiency occurs due to the following four types of feed combinations: (1) High Mo, generally above 100 ppm; (2) low Cu to Mo ratio, 2:1 or less; (3) Cu deficiency, below 5 ppm; and (4) high protein, 20-30% protein in fresh forage. The latter situation may be due to the high levels of sulfides produced from sulfuric amino acids during rumen fermentation.

The Cl content in plants is sufficient to meet the requirements of ruminants, since it ranged between 0.5 and 0.7 ppm in leaves. Furthermore, the content in each region is not significantly different. Chloride is the main anion in the extracellular fluid, 80-85% are inorganic and 15-20% are organic[31]. Chloride plays a significant role in the production of HCl in the abomasum and acid–base balance of the body [32]. It also plays a role in the absorption of amino acids and minerals, digestive proteins, and the regulation of osmotic pressure in the acid-base balance [4].

### Heavy metal content in soil, sea water, and mangrove plants

*A. marina* grows in extreme environments, where water and land experience ebbs and flows carrying various types of minerals. It is undeniable that plants will absorb non-essential minerals or even become toxic. From the analysis that has been performed, *A. marina* leaves in the coastal area of West Sumatra contain Hg at 0.04-0.08 ppm and Pb at 0.2 ppm. Meanwhile, heavy metal content in the leaves of each region was not significantly different. The content of Hg and Pb is relatively high in the soil, although it does not correlate with their solubility in water and absorption by *A. marina* plants. According to Kennady *et al*. [33], the heavy metal content in the soil is <7 and <200 ppm for Hg and Pb, respectively. Therefore, no heavy metal content in the study location exceeds the threshold. The mangrove leaves had a lower heavy metal content than the soil. This is because mangrove trees only require a small amount of these elements for growth [34].

Pb and Hg are toxic metals that have negative effects on livestock, even when consumed in small amounts. These metals inhibit the growth, productivity, and reproducibility of livestock and even cause death. Cows and sheep, which consume grass containing Pb at 5 mg/kg dry weight of grass/day, did not show any symptoms. However, pregnant ewes, which consumed grass containing Pb 30 mg/kg dry weight of grass/day for a long period of time, showed symptoms of poisoning, especially in conditions of starvation [33].

### Total phenol and tannin contents

The total phenol and tannin contents of *A. marina* leaves in the three sampling locations had shown a significant difference (Table-9). The number of ­polyphenols *Avicennia* in the Pariaman area was the highest compared with plants in other locations, reaching 24.51 mg GAE. Meanwhile, in the Padang and South Pesisir areas, plants share the same tendency and potential. Polyphenols are secondary metabolite compounds produced by plants to protect them from external disturbances. The high amounts of polyphenols observed in the Pariaman area were due to the growth of *A. marina* in the area, which often occurs due to storms and human touch.

**Table-9:** Polyphenol and Tannin content of *Avicennia* *marina* leaves grow in several coastal areas of West Sumatra.

Variable	Padang	Pariaman	Pesisir Selatan
Polyphenol (mg GAE/g)	13.02^a^±0.046	24.51^b^±0.056	10.03^a^±0.037
Tannin (%)	4.24±0.034	4.092^b^±0.012	1.40^a^±0.018

^a,b^Different superscripts mean significant difference in a row (p<0.05)

The content of secondary metabolites in plants is influenced by the environment, such as altitude, rainfall, and temperature. The influence of environmental factors interacts with genetic factors in the expression of secondary metabolites. Therefore, the production and excretion of secondary metabolites are influenced by temperature, light, soil, microorganisms, and nutritional status. Furthermore, secondary metabolite contents vary, depending on time and location. It is also related to variations in climatic and soil conditions, such as air, soil temperature, and soil moisture.

The tannin content of *A. marina* leaf samples extracted in the Padang and Pariaman areas was not significantly different at 4.24% and 4.092%, respectively; meanwhile, in the South Pesisir area, the tannin content was lower at 1.40%. The tannin content of *A. marina* leaves was still within tolerance limits for ruminants. Furthermore, tannins present in certain amounts will provide a positive value in increasing the digestibility of feed. Tannins are secondary metabolites synthesized and found in plants. They are classified as polyphenolic compounds with the ability to form complex compounds with other macromolecules. They also interact with proteins derived from feed, thereby reducing their availability for rumen microorganisms [35]. Therefore, this condition positively influences increasing forage digestibility, since proteins will be protected by tannins from the degradation of rumen microorganisms. These metabolites are mainly found in the post-rumen gastrointestinal tract. The tannin-protein binding complex can then be released at low pH in the abomasum, which enables protein degradation by the enzyme, pepsin. Therefore, the amino acids it contains are readily available to livestock. This explains the use of tannins for manipulating the level of protein degradation in rumens correlation between macromineral, micromineral, and heavy metals in soil to leaves showed at the Table-10. The mineral content of the soil has a positive correlation with the mineral content of the leaves. The higher the mineral content in the soil, the higher the mineral content in the leaves (Table-10).

**Table-10:** Correlation Between macromineral, micromineral, and heavy metals in soil to leaves.

	Leaves		

	Macrominerals	Micromineral	Heavy metals
Soil	0.969**	0.694**	0.787*

* Correlation significant at the 0.05,

** Correlation is significant at the 0.01

## Conclusion

This study showed that the minerals content of the soil in several mangrove areas of West Sumatra have a positive correlation with the minerals composition of *A. marina* leaves. Based on this study *A. marina* leaves contain complete minerals as forages for ruminant feed.

## Authors’ Contributions

GY, NJ, SS, BS, and RWWS: Formulated experimental design and experimental work at laboratory. GY: Drafted the manuscript and did data analysis under the guidance of NJ, SS, and BS. All the authors read and approved the final version of the manuscript.
